# MERS-CoV Antibody Responses 1 Year after Symptom Onset, South Korea, 2015

**DOI:** 10.3201/eid2307.170310

**Published:** 2017-07

**Authors:** Pyoeng Gyun Choe, R.A.P.M. Perera, Wan Beom Park, Kyoung-Ho Song, Ji Hwan Bang, Eu Suk Kim, Hong Bin Kim, Long Wei Ronald Ko, Sang Won Park, Nam-Joong Kim, Eric H.Y. Lau, Leo L.M. Poon, Malik Peiris, Myoung-don Oh

**Affiliations:** Seoul National University College of Medicine, Seoul, South Korea (P.G. Choe, W.B. Park, K.-H. Song, J.H. Bang, E.S. Kim, H.B. Kim, S.W. Park, N.-J. Kim, M.-D. Oh);; University of Hong Kong School of Public Health, Hong Kong, China (R.A.P.M. Perera, L.W.R. Ko, E.H.Y. Lau, L.L.M. Poon, M. Peiris)

**Keywords:** Middle East respiratory syndrome coronavirus, MERS, coronavirus, MERS-CoV, antibody, serology, kinetics, human, South Korea, viruses, zoonoses, neutralization, China

## Abstract

We investigated the kinetics of the Middle East respiratory syndrome coronavirus (MERS-CoV) neutralizing and spike protein antibody titers over the course of 1 year in 11 patients who were confirmed by reverse transcription PCR to have been infected during the outbreak in South Korea in 2015. Robust antibody responses were detected in all survivors who had severe disease; responses remained detectable, albeit with some waning, for <1 year. The duration of viral RNA detection (but not viral load) in sputum significantly correlated with the antibody response magnitude. The MERS S1 ELISA antibody titers correlated well with the neutralizing antibody response. Antibody titers in 4 of 6 patients who had mild illness were undetectable even though most had evidence of pneumonia. This finding implies that MERS-CoV seroepidemiologic studies markedly underestimate the extent of mild and asymptomatic infection. Obtaining convalescent-phase plasma with high antibody titers to treat MERS will be challenging.

Middle East respiratory syndrome (MERS) remains a disease of global public health concern for which no proven specific countermeasures are available. As of December 5, 2016, ≈1,800 laboratory-confirmed cases have been reported (*1*). MERS coronavirus (MERS-CoV) is an enzootic pathogen present in dromedary camels in many parts of the world, including the Middle East, Iran, Pakistan, and Africa (*2*,*3*). Zoonotic infections have been repeatedly reported on the Arabian Peninsula and have led to large nosocomial outbreaks. One notable example occurred in South Korea in 2015, initiated by a traveler returning home from the Arabian Peninsula (*4*). The infection in this traveler led to an outbreak of 186 cases and 36 deaths that had a substantial impact on the local economy. A cohort of 17 patients from this outbreak was intensively followed up to obtain detailed clinical, immunologic, and virologic characterization of their disease course (*5*,*6*). The kinetics of the serologic responses during the acute phase have already been reported, and they showed that robust but delayed antibody responses could be detected in patients who were more severely ill (*7*). Another study reported a significant linear correlation between the log_10_ viral loads and the serologic response in the acute phase of illness (*8*). The kinetics of the long-term serologic responses to MERS-CoV infections is poorly understood and remains of clinical interest. We report the results of a 1-year follow-up on the antibody responses in 11 of these patients.

## Material and Methods

### Patients

The acute-phase serologic responses of a cohort of 17 patients with reverse transcription PCR (RT-PCR)–confirmed MERS-CoV disease admitted to Seoul National University (SNU) Hospital in Seoul, South Korea; SNU Boramae Medical Center in Seoul; and SNU Bundang Hospital in Seongnam, South Korea, were previously reported (*7*). Nine of these patients had severe disease (defined as requiring supplemental oxygen or mechanical ventilation). The clinical, viral load, and cytokine profiles were previously reported (*5*,*6*).

We followed up 11 of these patients, 5 with severe disease (patients C, D, F, G, and I) and 6 with mild disease (patients K, L, M, N, O, and P), for 1 year. Their serum samples were collected at ≈6 months and ≈12 months after disease onset and used to investigate the long-term kinetics and duration of antibody responses that form the basis of this report. The clinical characteristics and early immunologic responses of the original and present cohorts of patients are summarized (Technical Appendix Table 1). The reasons for the lack of follow-up for the other 6 patients were transfer of care to another clinical unit (patient A), refusal of follow-up (patients J and Q), and death (patients B, E, and H). Patients B and E died during the acute phase of the illness, and patient H was discharged to receive rehabilitation care but was then given a diagnosis of aspiration pneumonia and died 2 months after disease onset. This study was approved by the Institutional Ethics Review Board of Seoul National University Hospital (approval no. 1506-093-681).

### Viruses

The human CoV-EMC/2012 strain was used for 50% tissue culture infectious dose assays, microneutralization assays, and plaque-reduction neutralization tests (PRNTs). A subset of serum samples was also tested with a strain from the outbreak in South Korea, MERS-CoV Hu/KOR/SNU1_035/2015.

### Serologic Tests

We heat inactivated serum samples for 30 min at 56°C before carrying out serologic tests. We performed the MERS-CoV PRNT (using a >90% plaque-reduction cutoff [PRNT_90_]), microneutralization test, and pseudoparticle neutralization test (ppNT) as described (*7*,*9*) (online Technical Appendix).

We used the MERS-CoV S1 ELISA kit (EI 2604-9601G; EUROIMMUN, Luebeck, Germany) for the detection of human IgG against MERS-CoV spike protein. We assayed serum samples in duplicate and performed the assay according to the manufacturer’s instructions. The assay included a calibrator, which defines the upper limit of the reference range for noninfected humans, and this upper limit served as the cutoff value. The assay was made semiquantitative by calculating the ratio of the extinction of the patient sample over the extinction of the calibrator. Ratios <0.8 were considered negative, ratios >1.1 were considered positive, and ratios >0.8 to <1.1 were considered borderline. We included known positive and negative control serum samples in all assays and denoted antibody titers in the reciprocal.

### Statistical Analysis

We calculated peak viral loads in sputum; PRNT_90_, ppNT, and microneutralization antibody titers; and MERS S1 ELISA optical density (OD) ratios of the 11 MERS patients by using descriptive statistics. Spearman correlation coefficients were calculated to correlate the peak viral loads in sputum during the acute phase of illness with serologic responses (PRNT_90_ antibody titers and MERS S1 ELISA OD ratios) at different times after disease onset. We also tested the correlation between duration of viral shedding and serologic responses at a 5% significance level. We excluded missing data from the main analyses. A sensitivity analysis was performed by imputing values from the most recent tests.

## Results

All 5 patients with severe disease, but only 2 (33%) of 6 with mild disease (p = 0.06 by Fisher exact test), had PRNT_90_ antibody titers >40 at the 1-year follow-up (Figure, panel A). PRNT_90_ antibody titers of patients C and F, who had acute-phase antibody titers of >320, declined >4-fold 1 year later. Patients D, G, K, and N who had acute phase peak antibody titers in the range of 80–160 only had <2-fold declines in titer. Patients C, D, F, G, I, K, and N (the 5 patients with severe disease and 2 of the 6 with mild disease who had PRNT_90_ antibody titers of >40 in their acute-phase serum samples [collected 21–50 days after disease onset]) continued to have detectable antibodies by PRNT_90_ (titer >1:40), ppNT (titer >1:40), microneutralization assay (titer >1:20), and S1 ELISA (ratio >1.1) 1 year after illness onset (Table; Figure, panel B). MERS antibody titers waned during the first 6 months after disease onset, especially in patients who had had high antibody titers. The waning of antibody titers between 6 months and 1 year after disease onset was less steep.

**Figure:**
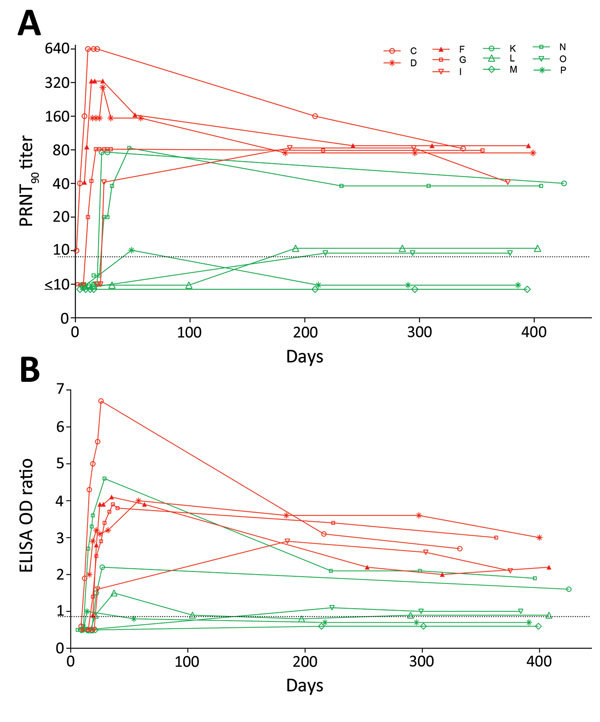
Middle East respiratory syndrome (MERS) coronavirus antibody titers in serially collected serum samples from 11 patients with reverse transcription PCR–confirmed symptomatic MERS, South Korea, 2015. PRNT_90_ titers (A) and MERS spike protein (S1) ELISA OD ratios (B) were determined at multiple time points 0 to >400 days after disease onset. The limit of detection was 10 for the PRNT, and the cutoff between negative and borderline samples for the S1 ELISA was an OD ratio of 0.8. Letters in key indicate patients; red indicates those with severe disease, and green indicates those with nonsevere disease. OD, optical density; PRNT_90_, >90% plaque-reduction neutralization test.

**Table:** Characteristics and serologic responses of patients from MERS-CoV outbreak by time after disease onset, South Korea, 2015*

Pt.	Severe disease	Peak viral load†	Corticosteroid use	Titers and ODs at various time points after disease onset														
				PRNT_90_ antibody titer				MERS-CoV spike ppNT				Microneutralization assay titer				MERS-CoV S1 ELISA OD‡		
21–50 d	180–248 d§	285–403 d§		21–50 d	180–248 d	285–403 d		21–50 d	180–248 d	285–403 d		21–50 d	180–248 d	285–403 d
C	Yes	6.1	No	640	160 (216 d)	80 (332 d)		640	160	80		320	160	40		6.2	2.9	2.5
D	Yes	8.2	Yes (11–23) ¶	160	80 (181 d)	80 (294 d), 80 (397 d)		160	160	80		80	80	80		2.6	3.0	2.5
F	Yes	4.8	Yes (14–16) ¶	320	80 (248 d)	80 (312 d), 80 (403 d)		320	320	40		160	40	40		3.6	1.7	1.5
G	Yes	7.2	No	80	80 (219 d)	80 (358 d)		80	80	160		80	80	80		3.4	2.9	2.5
I	Yes	5.2	No	NA#	80 (180 d)	80 (298 d), 40 (370 d)		40	40	40		40	40	40		1.1	2.4	1.6
K	No	6.7	No	80	NA	40 (420 d)		160	NA	40		40	NA	20		1.7	NA	1.1
L	No	4.6	No	<10	10 (192 d)	10 (285 d), 10 (403 d)		10	<10	<10		<10	<10	<10		1.0	0.3	0.4
M	No	5.4	No	NA**	<10 (209 d)	<10 (296 d), <10 (394 d)		<10	<10	<10		<10	<10	<10		0	0.1	0.1
N	No	6.6	No	80	40 (220 d)	40 (296 d), 40 (394 d)		40	80	40		80	40	20		4.1	1.6	1.4
O	No	8.2	No	NA	10 (218 d)	10 (294 d), 10 (379 d)		NA	<10	10		NA	<10	<10		NA	0.6	0.5
P	No	5.5	No	10	<10 (212 d)	<10 (290 d), <10 (386 d)		<10	<10	<10		<10	<10	<10		0.3	0.1	0.1

*MERS-CoV, Middle East respiratory syndrome coronavirus; NA, not assayed; OD, optical density; ppNT, pseudoparticle neutralization test; PRNT_90_, >90% plaque-reduction neutralization test; Pt., patient; S1, spike protein; upE, region upstream of the E gene. †Viral loads (log_10_ upE RNA copies/mL) were quantified from sputum samples. ‡MERS-CoV S1 ELISA OD ratios <0.8 were considered negative, ≥0.8 to <1.1 were considered borderline, and ≥1.1 were considered positive. §Exact day(s) after disease onset that serum was collected are indicated in parentheses. ¶Days after disease onset during which corticosteroids were used. #PRNT_90_ antibody titer was 40 on day 18. **PRNT_90_ antibody titer was 10 on day 16.

At 1 year after infection, the 4 patients who had mild disease (or who did not require supplemental oxygen or mechanical ventilation) all had negative results by microneutralization assay and S1 ELISA, but 1 was positive by ppNT (titer of 10) and 2 by PRNT_90_ (titer 1:10) (Table). Although designated as having mild disease, all of these patients, with 1 exception (patient P), had chest infiltrates on x-ray, indicating lung parenchymal pathology. 

The kinetics of antibody production seen with the PRNT_90_, ppNT, microneutralization test, and S1 ELISA were comparable (Table), suggesting that any of these tests could be used for detection of MERS-CoV antibodies in patients with past infection. One year after infection, all patients who had antibody titers of >20 by PRNT_90_ also had antibodies detectable by ppNT, microneutralization assay, and S1 ELISA. One patient (L) with a marginal PRNT_90_ titer of 1:10 was not positive by ppNT, microneutralization assay, or ELISA. At 1 year after infection, the correlation coefficients were 0.89 between PRNT_90_ and ppNT titers, 0.94 between PRNT_90_ and microneutralization assay titers, and 0.96 between PRNT_90_ and S1 ELISA titers.

The virus we used for serologic testing was the prototype MERS-CoV EMC clade A virus, and the virus that patients were exposed to (and that caused the outbreak in South Korea) was a clade B virus. To confirm that the neutralizing antibody titers against the clade A and B viruses were not significantly different, we tested 10 paired serum samples (from 5 MERS-CoV patients who had various levels of microneutralization antibody responses to EMC) by microneutralization assay using the clade A virus and a clade B virus from the outbreak in South Korea. The titers were similar (within a 2-fold dilution), confirming that the neutralizing epitopes of MERS-CoV are antigenically conserved (Technical Appendix Table 2).

The peak viral loads in sputum did not correlate with PRNT_90_ antibody titers or S1 ELISA OD ratios at the acute phase of illness, at ≈6 months after illness, or at ≈12 months after illness (Technical Appendix Table 3). However, we found strong positive correlations between duration of virus detection and antibody titers (as measured by the PRNT_90_ and S1 ELISA) at these time points (Technical Appendix Table 4). We defined the duration of virus detection as the day from symptom onset to negative PCR conversion. The median duration of virus shedding was 19 days (interquartile range 16.5–27.5 days). Only 2 of the patients (D and F) received corticosteroid therapy, and both had robust antibody responses (Table). However, because of the small number of patients given this treatment, evaluating its quality with a statistical analysis was not possible.

## Discussion

With this cohort of patients, we had previously reported that the severity of illness was associated with higher neutralizing antibody responses and ELISA ODs in the acute phase of illness (*7*). The analysis of antibody titers at ≈1 year after illness shows that these higher antibody titers continue to persist for at least 1 year. In the patients with the highest peak antibody titers, antibody titers waned during the first 6 months after infection but then stabilized over the next 6 months. This pattern of antibody production contrasts with the pattern reported for 1 patient with a microneutralization titer of 400, whose titers declined to an undetectable level within months by microneutralization test, immunofluorescent antibody (IFA) assay, and ELISA (*10*).

Patients K, L, M, N, O, and P were designated as having nonsevere disease because they were not given supplemental oxygen therapy, even though all of them, with the exception of patient P, had evidence of lung parenchymal disease. Only 2 of these patients (K and N) manifested robust antibody responses during the acute phase of the illness and early convalescence, and these titers were still present 1 year later. For the other patients, robust serologic antibody titers did not develop during the acute phase of the illness, and the patients remained seronegative or with marginal antibody titers at 1 year after infection. Because of the poor antibody response that resulted from symptomatic disease, persons with asymptomatic or mild infection without severe lung parenchymal disease are not expected to develop detectable MERS-CoV antibodies, a conclusion with implications for seroepidemiologic studies.

Three other studies have investigated the kinetics of long-term antibody persistence in patients with MERS-CoV illness. Survivors of a MERS-CoV outbreak in Jordan in April 2012 have been followed up with serologic testing at 13 months and 34 months after infection (*11*). These patients did not have RT-PCR–confirmed MERS-CoV infection, but they were considered to probably have MERS-CoV infections because they had positive serologic results together with an epidemiologic link to a confirmed MERS patient. Seven patients had symptomatic acute upper respiratory disease during the outbreak; of these, 5 had radiologic evidence of lower lung pathology, and 2 did not have chest radiologic examination data. All 7 patients had detectable antibodies by ELISA and IFA assay, and 6 of them had detectable antibodies by microneutralization test at 13 months and 34 months after infection with titers ranging from 20 to 80. However, only patients with robust serologic responses were included in this study (selection bias), and those without were excluded by definition.

In a study in Saudi Arabia, 9 patients with RT-PCR–confirmed MERS-CoV infection were followed up at 3 months, 10 months, and 18 months (for only 2 patients) after infection and tested by MERS-CoV S1 ELISA and IFA assay (*12*). These 9 patients (2 with severe pneumonia, 3 with nonsevere pneumonia, 1 with upper respiratory symptoms, and 3 with asymptomatic infections) were identified through contact tracing. All 5 patients with pneumonia were antibody positive by ELISA and IFA assay at 3 months after infection, 4 were positive by ELISA at 10 months after infection, and 3 were positive by IFA assay at 10 months after infection. In contrast, 0 of 4 patients with mild upper respiratory tract infection or asymptomatic infection were positive by ELISA or IFA assay at 3 or 10 months after infection. These data are comparable with our own, suggesting that milder infections are less likely to elicit serologic responses. However, that study did not provide data on virus neutralizing antibodies (*12*). A second study in Saudi Arabia reported that only 4 of 11 healthcare workers with real-time RT-PCR–confirmed MERS-CoV infection had detectable ELISA antibody titers ≈1 year after infection; of these 4 healthcare workers, 3 had detectable microneutralization antibody titers and only 1 had a high antibody titer (800) (*10*).

Waning antibody titers have also been demonstrated in patients with severe acute respiratory syndrome–CoV infection (*13*). Antibody titers peaked at 4 months after disease onset and declined to undetectable levels in 19% and 11% of serum samples by IgG test and microneutralization test, respectively, 30 months after infection. Geometric mean microneutralization antibody titers dropped from 1,232 at month 4 to 32 at month 30 after infection and remained at that level until month 36 (*13*). In volunteers experimentally infected with human CoV 229E, neutralizing antibody titers peaked at 3 weeks after infection and fell considerably by 12 weeks after infection, declining close to baseline levels by 1 year (*14*). However, human CoV 229E infects mainly the upper respiratory tract, unlike the pathogens of severe acute respiratory syndrome and MERS, which are more invasive of the lung parenchyma and often disseminate systemically.

Convalescent-phase plasma therapy has been proposed as a treatment option for the acute respiratory diseases, like MERS, that do not have specific antimicrobial treatments available (*15*,*16*). Our data indicate that plasma with high MERS-CoV antibody titers are only likely to be available from patients who have recovered from severe MERS disease, and these titers substantially wane within the first 6 months of illness, although lower levels of MERS-CoV antibodies are maintained over longer periods. To use convalescent-phase patient plasma for treatment, it will be necessary to assess the antibody titer of potential donors before collection to ensure good antibody titer. A neutralization test is likely to be the optimal assay for assessing plasma used for therapy, but because our data indicated that the S1 ELISA correlates well with neutralization titers, the S1 ELISA might be a suitable screening test for selecting persons for plasma donation.

One limitation of this work is the virus used for neutralization tests. The clade A virus MERS-CoV EMC was used for assays, and the virus the patients were infected with and that caused the outbreak in South Korea was a clade B virus (the dominant virus clade currently circulating in the Arabian Peninsula). It has been shown that clade A and B viruses do not differ antigenically at the neutralization epitope, although they are genetically distinct (*17*,*18*). Using a subset of serum samples from this study, we confirmed that neutralizing antibody titers obtained with the clade A EMC virus were similar to those obtained with a clade B virus strain from the outbreak in South Korea.

In conclusion, our findings support and extend the research of others. We suggest that serologic tests for MERS-CoV antibodies can only identify some of the patients who have had MERS-CoV infections. Serologic responses to this virus are variable, not robust, and often undetectable when disease is mild. Thus, MERS-CoV seroepidemiologic studies will only detect a fraction of infections that are occurring in a population and will probably markedly underestimate the extent of mild infection that is taking place. Our findings also show that the MERS S1 ELISA is as good as neutralization tests at detecting antibodies a year after infection, but positive ELISA results do require confirmation with neutralization tests if false positives are to be avoided in seroepidemiologic assays (M. Peiris, unpub. data). Convalescent-phase plasma can be harvested for many months to a year after disease from patients surviving MERS-CoV infection, but plasma with a high antibody titer is only likely to be obtained during the first few months of convalescence from persons who had severe disease. Because patients during this time frame are likely to be frail, this approach will be challenging.

Technical Appendix Description of methods, characteristics of a cohort of patients who became infected with Middle East respiratory syndrome coronavirus (MERS-CoV) during the outbreak in South Korea in 2015, comparison of patient antibody titers tested with clade A and B MERS-CoV isolates, correlation between peak viral load and MERS-CoV serologic responses, and correlation between shedding duration and MERS-CoV serologic responses.
