# Supplementary material for: MERS-CoV Antibody Responses 1 Year after Symptom Onset, South Korea, 2015
Source: Emerg Infect Dis. 2017 Jul;23(7):1079–84. doi: 10.3201/eid2307.170310 (PMC5512479; doi:10.3201/eid2307.170310)
Supplement: Technical Appendix — Description of methods, characteristics of a cohort of patients who became infected with Middle East respiratory syndrome coronavirus (MERS-CoV) during the outbreak in South Korea in 2015, comparison of patient antibody titers tested with clade A and B MERS-CoV isolates, correlation between peak viral load and MERS-CoV serologic responses, and correlation between shedding duration and MERS-CoV serologic responses. [file 17-0310-Techapp-s1.pdf]

Article DOI: https://dx.doi.org/10.3201/eid2307.170310

MERS-CoV Antibody Responses 1 Year after Symptom Onset, South Korea, 2015

Technical Appendix

Methods

The plaque-reduction neutralization test (PRNT) was performed in duplicate in a biosafety level 3 facility by using 24-well tissue culture plates (TPP Techno Plastic Products AG, Trasadingen, Switzerland). Serial dilutions of serum samples were incubated with 50–70 plaque-forming units of virus for 1 h at 37°C. The virus-serum mixtures were added on to Vero cell monolayers and incubated 1 h at 37°C in 5% CO_2 incubator. Then the plates were overlaid with 1% agarose in cell culture medium and incubated for 3 days when the plates were fixed and stained. Antibody titers were defined as the highest serum dilution that resulted in $\geq$90% ($PRNT_{90}$) reduction in the number of plaques (*1*).

For the microneutralization assay, 2-fold dilutions of serum samples were incubated with an equal volume of 200 tissue culture infectious dose 50s of Middle East respiratory syndrome coronavirus (MERS-CoV) (*2*). After 1 h incubation at 37°C, the virus-serum mixture was added in quadruplicate to preformed Vero cell monolayers in 96-well microtiter plates (TPP Techno Plastic Products AG). Cytopathic effect was observed at 3 days postinfection. The highest serum dilution that completely protected the cells from cytopathic effect in $\geq$2 of the 4 wells was taken as the neutralizing titer. Virus back titrations were carried out in each assay to confirm that the virus challenge dose was within expected range.

The virus pseudoparticle neutralization test (ppNT) was performed as previously described (*2*). Lentivirus pseudoparticles containing codon optimized MERS-CoV (EMC strain) spike protein and a luciferase reporter gene were incubated with serially diluted serum samples for 1 h at 4°C and then added to Vero E6 cells (CRL-1586; American Type Culture Collection, Manassas, VA, USA) in triplicate. Residual virus replication was assayed by the luciferase counts at 2 days postinfection. The highest serum dilution giving a 90% reduction of luciferase activity was regarded as the ppNT antibody titer.

References

1. Park WB, Perera RA, Choe PG, Lau EH, Choi SJ, Chun JY, et al. Kinetics of serologic responses to MERS coronavirus infection in humans, South Korea. Emerg Infect Dis. 2015;21:2186–9. PubMed http://dx.doi.org/10.3201/eid2112.151421

2. Perera RA, Wang P, Gomaa MR, El-Shesheny R, Kandeil A, Bagato O, et al. Seroepidemiology for MERS coronavirus using microneutralisation and pseudoparticle virus neutralisation assays reveal a high prevalence of antibody in dromedary camels in Egypt, June 2013. Euro Surveill. 2013;18:20574. PubMed http://dx.doi.org/10.2807/1560-7917.ES2013.18.36.20574

Technical Appendix Table 1. Characteristics and $PRNT_{90}$ titers of the patients from the MERS-CoV outbreak, South Korea, 2015*

Patient	Sex/age, y	Underlying disease	CXR infiltrates	Oxygen therapy	Mechanical ventilation	Corticosteroid therapy	Antiviral use	Outcome (day post disease onset)	Included in present study	$PRNT_{90}$ antibody titer 21–50 days post disease onset
A	M/38	–	Yes	Yes	Yes	Yes	Yes	Transfer (61)	No	80
B	M/65	–	Yes	Yes	Yes	Yes	Yes	Died (142)	No	≥320
C	M/55	–	Yes	Yes	Yes	No	Yes	Discharge (26)	Yes	640
D	M/35	Bacterial pneumonia	Yes	Yes	Yes	Yes	Yes	Discharge (33)	Yes	160
E	F/79	CHD, dementia, CKD, bladder cancer	Yes	Yes	Yes	Yes	Yes	Died (17)	No	NA (<10 on day 16)
F	M/55	DM, COPD, lung abscess	Yes	Yes	No	Yes	No	Discharge (31)	Yes	320
G	M/56	–	Yes	Yes	No	No	Yes	Discharge (40)	Yes	80
H	M/71	DM, CVA, aspiration pneumonia	Yes	Yes	No	No	No	Discharge (38)	No	160
I	F/77	DM, asthma	Yes	Yes	No	No	No	Discharge (18)	Yes	NA (40 on day 18)
J	M/76	DM, CHD	Yes	No	No	No	No	Discharge (28)	No	40
K	M/59	CHD	Yes	No	No	No	Yes	Discharge (19)	Yes	80
L	F/56	–	Yes	No	No	No	No	Discharge (21)	Yes	<10
M	M/56	DM, CHD, CLD, TB	Yes	No	No	No	No	Discharge (16)	Yes	NA (<10 on day 16)
N	F/54	–	Yes	No	No	No	No	Discharge (21)	Yes	80
O	M/46	–	Yes	No	No	No	No	Discharge (12)	Yes	NA
P	M/35	–	No	No	No	No	Yes	Discharge (14)	Yes	10
Q	M/52	DM, liver abscess	Yes	No	No	No	Yes	Discharge (21)	No	160

*CHD, coronary heart disease; CKD, chronic kidney disease; CLD, chronic lung disease; COPD, chronic obstructive pulmonary disease; CVA, cerebrovascular accident; CXR, chest X-ray; DM, diabetes mellitus; NA, not applicable; $PRNT_{90}$, ≥90% plaque-reduction neutralization test; TB, tuberculosis.

Technical Appendix Table 2. MERS-CoV antibody titers in patient serum samples*

Patient ID	Day serum collected after disease onset	Reciprocal MERS-CoV MN antibody titer	
		MERS-CoV EMC	MERS-CoV Hu/KOR/SNU1_035/2015
F	58	80	160
F	403	40	40
M	16	<10	<10
M	394	<10	<10
L	99	<10	<10
L	403	<10	<10
O	8	<10	<10
O	379	<10	<10
I	18	20	40
I	298	20	40

*Patient titers were assessed with prototype strain EMC and a virus isolate Hu/KOR/SNU1_035/2015 from the outbreak in South Korea in 2015. ID, identification; MERS-CoV, Middle East respiratory syndrome coronavirus; MN, microneutralization.

Technical Appendix Table 3. Spearman correlation between peak viral loads in the acute phase of illness and serologic responses at different time points post disease onset*

Time post disease onset	$PRNT_{90}$		ELISA OD ratio	
	Spearman correlation	p value	Spearman correlation	p value
Acute phase†	0.20	0.56	0.13	0.71
≈6 mo	0.17	0.64	0.45	0.19
≈1 y	0.17	0.62	0.37	0.26

*Peak viral load (log_{10} UpE virus RNA copies/mL) was measured in sputum. All correlations were not statistically significant and changed by <0.1 with imputed values from nearest testing occasions. OD, optical density; $PRNT_{90}$, ≥90% plaque-reduction neutralization test; upE, region upstream of the E gene.

†Peak antibody titer during the acute phase was used in these analyses.

Technical Appendix Table 4. Spearman correlation between durations of viral shedding and serologic responses at different time points post disease onset*

Time post disease onset	$PRNT_{90}$		ELISA OD ratio	
	Spearman correlation	p value	Spearman correlation	p value
Acute phase†	0.74	0.010	0.77	0.006
≈6 mo	0.74	0.015	0.79	0.007
≈1 y	0.79	0.004	0.80	0.003

*All correlations were statistically significant and changed by <0.06 with imputed values from nearest testing occasions. OD, optical density; $PRNT_{90}$, ≥90% plaque-reduction neutralization test.

†Peak antibody titer during the acute phase was used in these analyses.
